# Differentiating Urgent and Emergent Causes of Acute Red Eye for the Emergency Physician

**DOI:** 10.5811/westjem.2016.12.31798

**Published:** 2017-03-03

**Authors:** Christopher J. Gilani, Allen Yang, Marc Yonkers, Megan Boysen-Osborn

**Affiliations:** * University of California, Irvine School of Medicine, Irvine, California; † Western University of Health Sciences, College of Osteopathic Medicine of the Pacific, Pomona, California; ‡ University of California, Irvine, Medical Center, Gavin Herbert Eye Institute, Department of Ophthalmology, Irvine, California; § University of California, Irvine, Medical Center, Department of Emergency Medicine, Orange, California

## Abstract

Patients commonly present with an acute red eye to the emergency department (ED). It is important to distinguish between benign and sight-threatening diagnoses. Here we provide a comprehensive overview on the acute red eye in the ED.

## INTRODUCTION

Emergency physicians (EP) must be knowledgeable in the evaluation of the acute red eye. For the purposes of this review, the acute red eye refers to a patient with conjunctival and/or scleral redness. The differential diagnosis ranges from routine (subconjunctival hemorrhage) to immediately sight-threatening diagnoses (acute angle closure glaucoma [AACG] or endophthalmitis). Asking key historical questions and performing a complete ocular examination will help to distinguish whether emergent, urgent, or as-needed ophthalmologic follow up is necessary. Here we discuss key historical and physical examination features in the workup of the acute red eye. We provide a comprehensive overview of the differential diagnosis for the patient who presents to the emergency department (ED) with an acute red eye.

## HISTORICAL FEATURES

### Pain or Photophobia

Pain and/or photophobia are important features in distinguishing between minor and serious ophthalmologic diagnoses. Mild irritation or foreign body sensation may be present in minor diagnoses (conjunctivitis, episcleritis).^1^,^2^ Early viral keratitis, however, may present with irritation only. It is important to perform a thorough skin and fluorescein examination in these patients. Physicians should take caution in any patient who has pain or photophobia, as these can be signs of more serious diagnoses (AACG, bacterial keratitis, scleritis, anterior uveitis). Photophobia can either be direct, consensual, or both. Direct photophobia refers to pain with light shone in the affected eye; whereas, consensual photophobia refers to pain with light shone in the unaffected eye. Consensual photophobia, though a subjective finding, is suggestive of iritis (anterior uveitis) over superficial corneal processes.^3^ Corneal abrasions may present with severe pain, but the pain typically subsides in 24–48 hours and patients will have a characteristic lesion on fluorescein examination.^4^ Patients with corneal abrasions from contact lenses should routinely see an ophthalmologist within 24–48 hours, especially if symptoms have not improved.^4^

### Associated Symptoms

The EP should determine if the patient has any associated symptoms, such as headache or vomiting, concerning for AACG.^5^ Symptoms of an upper respiratory tract infection are often associated with viral conjunctivitis.^1^

### History of Trauma, Exposure, or Surgery

A history of minor trauma should raise suspicion for a corneal abrasion or subsequent infectious keratitis.^4^,^6^ Physicians should be concerned for an ocular foreign body in metal workers or ultraviolet (UV) keratitis in patients with exposure to the sun or occupational UV light.^7^ A history of moderate or major trauma should raise suspicion for globe rupture or traumatic iritis.^8^,^9^ The EP should strongly consider endophthalmitis in a patient with recent ophthalmologic surgery.^10^ Chemical burns or chemical conjunctivitis are the result of ocular chemical exposure; identification of the chemical content of the exposure and possible acidity or basicity may aid therapy.

### Risk Factors

Episcleritis, scleritis, and anterior uveitis are associated with autoimmune and rheumatologic conditions.^2^ Patients with a history of contact lens use are at an increased risk for infectious keratitis.^11^ Medication history may also guide diagnosis; for example, anticoagulants are associated with subconjunctival hemorrhage, while topiramate is associated with angle closure.

## PHYSICAL EXAMINATION FEATURES

### Skin and Lid Examination

In a patient with an acute red eye, herpetic lesions on the skin warrant further investigation for herpes or varicella keratitis by fluorescein and slit lamp examination.^8^ If there is confirmed or high suspicion for herpes or varicella keratitis patients should be started on oral or topical antivirals in the ED. The patient should have urgent (24–48 hours) follow up with ophthalmology to determine the extent of ocular involvement. If antiviral treatment is not initiated, ophthalmologic follow up or consultation should be within 12 hours. Erythema or edema of the skin should raise suspicion for periorbital cellulitis, dacrocystitis, stye, or blepharitis, which may have associated conjunctivitis. More serious causes of an acute, red painful eye with periorbital edema and erythema are orbital cellulitis and cavernous sinus thrombosis, which may present with pain on eye movement or ophthalmoplegia. It is also important to examine underneath the lid (“flipping the lid”) in patients with a corneal epithelial defect (positive fluorescein staining often vertically oriented) to ensure that there is no retained foreign body, causing repetitive trauma to the eye.

### Visual Acuity

An assessment of visual acuity (VA) should be performed in all patients presenting with ocular complaints. The patient should wear his/her own corrective lenses for the exam with distance or near correction as necessary.^9^ If the patient does not have corrective lenses, a practitioner can perform a VA with pin holes to compensate for refractive error. When administering a visual acuity exam, patients should be encouraged to give their best “guess” for each line. For patients with significant discomfort due to a corneal abrasion, the VA should be checked after application of topical anesthetics. An acutely decreased visual acuity should raise a high suspicion for a vision-threatening process, such as AACG or endophthalmitis.

### Response to Topical Anesthetic

Instillation of proparacaine or other anesthetic eye drops should significantly improve symptoms if the pain is secondary to a lesion at the corneal or conjunctival surface, such as a corneal abrasion. Improvement of pain following topical anesthetic administration is reassuring; however, corneal ulcers/bacterial keratitis, foreign bodies, and viral keratitis must still be considered. While some studies have supported the practice of discharging patients home with a short course of topical anesthetics,^12^–^14^ we do not recommend this as routine practice, as their use is toxic to the corneal epithelium and can potentially result in severe complications.^15^,^16^

### Response to Phenylephrine

Although we do not routinely instill phenylephrine drops to all patients with an acute red eye, the response to phenylephrine is useful in distinguishing between episcleritis and scleritis.^2^ The redness of episcleritis should improve with instillation of phenylephrine, as the episcleral vessels constrict, but the redness of scleritis should not improve.^2^ Phenylephrine should be instilled only after accurate normal intraocular pressure (IOP) has been determined, so as to not exacerbate AACG.^17^

### Slit Lamp Examination

A slit lamp examination is necessary to identify cells and flare in the anterior chamber, as this is a sign of an acute inflammatory process, such as anterior uveitis or bacterial keratitis.^11^ While up to 75% of patients with bacterial keratitis will not have anterior chamber inflammation,^18^ cell and flare in the anterior chamber warrant urgent ophthalmologic consultation.^11^ The EP can assess for anterior chamber inflammation at the slit lamp by setting the slit beam at a small 1×1 mm beam and projecting it at an oblique angle through the anterior chamber. Inflammation is characterized by the presence and density of circulating immune cells (cell) and a foggy appearance to the slit beam (flare) caused by protein leaking into the anterior chamber through inflamed vessels. The slit lamp will also identify a corneal infiltrate associated with bacterial or fungal keratitis.

### Fluorescein Examination

In conjunction with the slit lamp examination, fluorescein will identify a corneal epithelial defect, such as a corneal abrasion or a corneal defect associated with a microbial keratitis infiltrate. UV keratitis can present with diffuse punctate staining. Branching lesions with end bulbs that brightly stain with fluorescein are typical of herpes simplex virus (HSV).^19^ Small, non-staining vesicles may be the only finding during the first 24 hours of HSV infection, or in patients who are immunocompromised.^19^ Highly branched lesions without end bulbs are typical of varicella zoster virus (VZV), and these stain less brightly with fluorescein.^19^ In addition to VZV, these “pseudodendrites” can be caused by neurotrophic epitheliopathy and Acanthamoeba, but these diagnoses are beyond the scope of the EP.^19^

In a patient with a normal lid examination (or for patients with vesicles on their lid examination), the presence or absence of pain and/or photophobia, response to phenylephrine and topical anesthetics, intraocular pressure, slit lamp examination (including fluorescein), and visual acuity are the most helpful historical and physical findings in distinguishing between mild and serious processes.

## DIFFERENTIAL DIAGNOSIS FOR THE ACUTE RED EYE WITH NORMAL LIDS

### Subconjunctival Hemorrhage

Subconjunctival hemorrhage (SH) is defined as the presence of heme under the conjunctiva, secondary to a ruptured conjunctival blood vessel.^8^ Risk factors for SH include trauma, straining (coughing, sneezing, vomiting, Valsalva), conjunctivitis, chronic health conditions (diabetes, hypertension), and coagulopathy.

- Pain: None
- Photophobia: None
- Response to topical anesthetic: Not applicable
- Response to phenylephrine: None
- Visual Acuity: Normal
- Pupils: Normal
- Anterior chamber: Clear
- Fluorescein: No uptake

It is important to consider globe rupture in patients with a history of blunt or penetrating trauma or 360-degree bullous hemorrhage. The EP may consider checking an INR in patients on warfarin. One should also consider non-accidental trauma in a child with SH and no history of vomiting or straining.^20^,^21^ Patients with subconjunctival hemorrhage may be reassured and advised to use topical lubrication as needed. Patients may be referred to a primary care doctor for routine follow up of any chronic health conditions.

### Conjunctivitis

Conjunctivitis is defined as infectious or non-infectious inflammation of the bulbar and palpebral conjunctiva.^1^ Patients typically have a mild burning sensation, tearing, discharge, and associated viral symptoms. Conjunctivitis can be viral, bacterial, or allergic. While purulent/mucopurulent discharge is more typical of bacterial conjunctivitis and watery discharge is typical for viral conjunctivitis, this distinction is not entirely reliable.^22^ One study found that the combination of bilateral eye mattering (crusting), lack of itching, and lack of prior history of conjunctivitis was most predictive of bacterial conjunctivitis.^22^ Viruses cause the majority of cases of conjunctivitis, most commonly adenovirus.^22^ Acute bacterial conjunctivitis may be caused by *Staphylococcus aureus, Streptococcus pneumoniae, Haemophilus influenza, Neisseria gonorrhea, Chlamydia trachomatis,* or diphtheria. *Neisseria gonorrhea* causes a hyperacute bacterial conjunctivitis with copious purulent discharge.^22^

- Pain: Minimal to none
- Photophobia: None
- Response to topical anesthetic: Reduction in irritation
- Response to phenylephrine: Mild improvement in redness
- Anterior Chamber: Clear
- Pupils: Normal
- Visual Acuity: Normal
- Fluorescein: No uptake

Patients with allergic conjunctivitis can be treated with topical antihistamines. Viral conjunctivitis can be treated with supportive care and preservative-free artificial tears up to eight times per day. Viral conjunctivitis is highly contagious one to two weeks from onset. Contact precautions are recommended; sterilization of the patient encounter room should be performed after the visit.

Topical antibiotics for bacterial conjunctivitis shorten the duration of disease, but the disease itself is usually self-limited.^22^ Furthermore, topical antibiotics may cause side effects such as worsening eye irritation and community anti-microbial resistance.^22^ Patients with hyperacute bacterial conjunctivitis secondary to *N. gonorrhea* should be treated for both *N. gonorrhea* and *C. trachomatis* with ceftriaxone and azithromycin or doxycycline. Appropriate treatment is necessary to avoid corneal involvement and perforation.^22^

Patients with viral or bacterial conjunctivitis can follow up with primary care, but contact lens users should have close follow up to ensure that their “conjunctivitis” is not an early bacterial keratitis. One should question the diagnosis of conjunctivitis if the palpebral conjunctiva is not involved. One should similarly question the diagnosis if the patient is having pain or photophobia, as these symptoms should raise suspicion for bacterial keratitis or uveitis.

### Episcleritis

Episcleritis is defined as idiopathic inflammation of the episclera, which is the vascularized tissue between conjunctiva and sclera.^2^ Episcleritis risk factors include female gender (70%), age (fifth decade of life), and systemic autoimmune conditions.^2^ Redness is usually focal in the interpalpebral zone (the area visible when the eye is open).

- Pain: Mild irritation is possible; chronic or nodular episcleritis may have pain^2^
- Photophobia: None
- Response to topical anesthetic: May improve irritation
- Response to phenylephrine: Resolution of episcleral redness after 10–15 minutes (key feature)
- Visual Acuity: Normal
- Pupils: Normal
- Anterior Chamber: Clear
- Fluorescein: No uptake

The key feature in distinguishing between episcleritis and scleritis is the patient’s response to phenylephrine. The vessels in episcleritis will constrict and the eye redness will improve; this is not true of scleritis. Additionally, the inflamed vessels of episcleritis will move with gentle pressure from a cotton-tipped applicator. Patients with episcleritis are treated with topical lubricants and oral non-steroidal anti-inflammatory drugs.^23^ Patients can follow up with primary care for continued management and for workup of any underlying cause. Patients should be given return precautions for symptoms of scleritis (worsening pain).

### Anterior Scleritis

Anterior scleritis is defined as scleral inflammation that is frequently associated with autoimmune systemic disease.^2^ Fifty percent of patients with anterior scleritis have associated autoimmune, systemic disease (rheumatoid arthritis, granulomatosis with polyangiitis, formerly known as Wegener’s granulomatosis), while 4–10% have associated infectious processes.^2^ There are three forms of anterior scleritis: diffuse, nodular, and necrotizing, the latter of which usually causes the most severe pain and has the worst outcome. The sclera may have a typical blush in natural light as uveal tissue may be apparent through a thin and inflamed sclera.^24^

- Pain: Gradual onset, severe, boring, and piercing eye pain. Pain is worse at night, with extraocular movements, and may radiate to the face^2^,^24^
- Photophobia: May be present
- Response to topical anesthetic: Should not improve pain
- Response to phenylephrine: Redness does not improve
- Visual Acuity: Normal or decreased, depending on extent of the disease
- Pupils: Normal
- Anterior Chamber: Clear
- Fluorescein: May show peripheral keratitis, which is more common in the necrotizing form.^2^

Patients with anterior scleritis should be referred emergently to ophthalmology to initiate treatment and to prevent scleral melting.^9^,^25^ If there is excessive scleral thinning, patients are at risk for perforation and an eye shield should be placed.

### Anterior Uveitis/Iritis

Anterior uveitis is defined as idiopathic inflammation of the uvea (iris, choroid, and/or ciliary body), causing redness and pain. Risk factors include systemic diseases (spondyloarthropathies), infectious processes (syphilis, tuberculosis, Lyme disease, toxoplasmosis, herpesviruses, cytomegalovirus), and certain drugs (rifabutin, cidofovir, sulfas, moxifloxacin).^26^ Patients present with pain, diffuse redness pronounced at the limbus (ciliary flush), consensual photophobia, tearing, and possibly decreased vision.

- Pain: Moderate to severe
- Photophobia: Consensual photophobia (key feature)
- Response to topical anesthetic: Should not improve pain
- Response to phenylephrine: Redness does not improve
- Visual Acuity: Normal or decreased
- Pupils: Constricted or irregular
- Anterior Chamber: Cells and flare present
- Fluorescein: May reveal dendrites if the underlying cause is HSV.

The treatment for anterior uveitis is topical steroids, although this should only be done in conjunction with ophthalmologic consultation, since topical steroids may worsen the prognosis for patients with HSV keratitis. Patients may also be treated with dilating drops to help to prevent scarring of the iris to the lens (synechiae). Patients must follow up with ophthalmology within 24 hours to control symptoms, limit inflammatory consequences, and to consider lab work for an underlying cause.

### Acute Angle Closure Glaucoma

AACG is defined as closure (or narrowing) of the anterior chamber angle, causing elevated intraocular pressure and eventual optic nerve damage.^5^,^27^ Risk factors include increased age, female gender (three times more common), Asian ethnicity, shallow anterior chamber, hyperopia, and certain medications (topiramate or sulfa).^27^ Patients typically present with headache, nausea, vomiting, halos around lights, photophobia, blurred vision, and pain. Eye redness is diffuse, with characteristic ciliary flush.

- Pain: Moderate to severe
- Photophobia: Present
- Response to topical anesthetic: Should not improve pain
- Response to phenylephrine: Instillation of phenylephrine may exacerbate condition and should not be given^17^
- Visual Acuity: Decreased
- Pupils: Mid-sized or dilated, non-reactive pupil^17^
- Anterior Chamber: Shallow
- Fluorescein: No uptake

The diagnosis of AACG is confirmed with elevated intraocular pressure, which may be elevated as high as 60 mmHg. AACG must be treated early to avoid optic nerve ischemia. Treatment includes topical parasympathomimetics (pilocarpine 2%; avoid anything higher than 2%), topical beta-blocker (0.5% timolol; caution in asthmatics, COPD patients, and patients with heart block), carbonic anhydrase inhibitors (acetazolamide, 500 mg IV; avoid in sickle cell patients and possibly in sulfa allergy patients), and alpha agnoists (brimonidine 0.1%; avoid in patients on monoamine oxidase inhibitors). Practitioners should avoid apraclonidine, as it can lead to dilation. Ophthalmologic consultation should be sought emergently for continued management recommendations and definitive treatment, usually with laser iridotomy.

### Corneal Abrasion/Corneal Foreign Bodies

Defects to the corneal epithelium from abrasions or foreign bodies can cause irritation, pain, tearing, and photophobia.^4^ Risk factors include trauma, contact lens use, male gender, young age, and construction or manufacturing job without the use of eye protection. For corneal abrasions, moderate or severe pain is common, but it usually lasts less than 24–48 hours.^4^

- Pain: Moderate to severe, lasts less than 48 hours
- Photophobia: Present
- Response to topical anesthetic: Should significantly improve pain
- Response to phenylephrine: Redness improves
- Visual Acuity: May be decreased if the defect is in the visual axis
- Pupils: Normal
- Anterior Chamber: Normal
- Fluorescein: Uptake at the site of the corneal abrasion (corneal epithelial defect).

Corneal abrasions are treated with lid eversion to exclude foreign body, lubricating ointment or drops, and topical antibiotics (polymixin B, trimethoprim or polysporin; quinolones for contact lens wearers). Contact lens wearers should follow up with ophthalmology within 48 hours. If there is concern for a corneal ulcer or if the pain is not improving within 24 hours, patients should be referred to ophthalmology emergently. A corneal foreign body requires removal of foreign body by an ophthalmologist or EP as soon as possible. A Seidel’s test should be performed if there is concern for corneal laceration or globe rupture. A Seidel’s test is performed by placing fluorescein dye gently against the bulbar conjunctiva; any disruption of epithelial cells will show positive staining.^28^

### Bacterial or Fungal Keratitis/Corneal Ulcer

**Figure 8:**
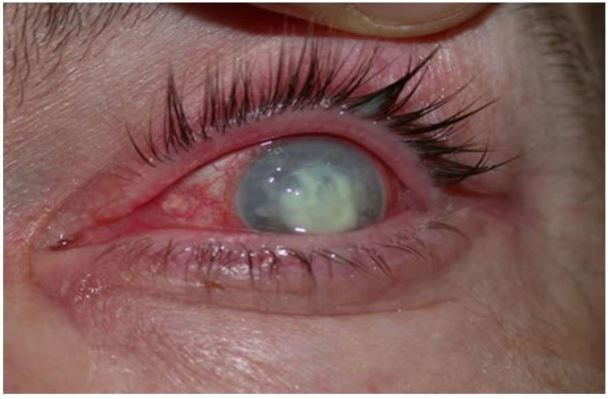
Corneal ulcer. Image courtesy of Andrew Pearson, MA, MRCP.

Bacterial or fungal keratitis is a corneal epithelial defect with stromal haze due to microorganisms.^6^ Risk factors include the following: contact lens use,^18^,^29^ agricultural work,^11^ eye trauma (including corneal abrasion), use of corticosteroids, systemic diseases (diabetes), prior ocular surgery, and chronic ocular surface disease.^11^ It is rare to develop bacterial or fungal keratitis without risk factors.^18^ *Staphylococcus aureus,* coagulase negative staphylococci, and *Pseudomonas aeruginosa* are commonly isolated organisms in bacterial keratitis.^18^,^30^ Patients present with diffuse redness of the eye accompanied by significant pain, tearing, discharge, and photophobia. The exam may mimic that of conjunctivitis, so it is important to have a high index of suspicion for microbial keratitis in patients with pain and/or risk factors. While Figure 8 shows a very large corneal ulcer, the presentation may be much more subtle, with about 40% of patients presenting with lesions smaller than 5 mm^2^.^18^

- Pain: Moderate to severe
- Photophobia: Present
- Response to topical anesthetic: May improve pain
- Response to phenylephrine: May improve redness
- Visual Acuity: May be decreased if the defect is in the visual axis
- Pupils: Normal
- Anterior Chamber: Cell and flare in 25% of patients; patients may have a frank hypopyon^18^
- Fluorescein: Uptake at the associated corneal epithelial defect

Treatment includes fortified topical antibiotics. Patients should follow up with ophthalmology within 24 hours (or sooner, depending on the severity). The complications of microbial keratitis include corneal perforation and extension into the visual axis.

### Endopthalmitis

Endopthalmitis is defined as a bacterial or fungal infection involving the vitreous and/or aqueous humor.^10^ Risk factors include eye surgery (cataract surgery has 0.1% risk), penetrating ocular trauma, corneal infection, intravitreal injections, and hospitalization with central venous access, total parenteral nutrition, or broad spectrum antibiotics.^10^ Endophthalmitis usually occurs 2–7 days post-operatively or 12–24 hours after trauma.

- Pain: Moderate to severe
- Photophobia: Present
- Response to topical anesthetic: No improvement
- Response to phenylephrine: Minimal improvement
- Visual Acuity: Decreased
- Pupils: May have afferent pupillary defect^31^
- Anterior Chamber: Commonly associated with hypopyon^10^
- Fluorescein: May diagnose the inciting traumatic lesion

Ophthalmology should be consulted immediately. If the patient’s vision is “hand motion” or better, then the patient is treated with intravitreal injection of antibiotics by ophthalmology. If the patient’s vision is “light perception” or worse, then the patient will need an emergent vitrectomy with ophthalmology if there is potential for vision loss.

### Viral Keratitis

Viral keratitis is defined as corneal inflammation caused by herpes simplex (HSV-1 most commonly), varicella zoster, or adenovirus (adenovirus 8, 19, 37 causing epidemic keratoconjunctivitis, EKC) characterized by pain, tearing, photophobia, and corneal epithelial defects.^32^ Patients at risk for VZV keratitis typically have a the characteristic vesicular rash in the V1 (ophthalmic) branch of the trigeminal nerve. It is classically taught that a lesion on the nose, indicating involvement of the nasociliary branch of the ophthalmic nerve precedes ocular involvement of VZV, although this is not sensitive or specific.^33^

- Pain: Present
- Photophobia: Present
- Response to topical anesthetic: May improve pain
- Response to phenylephrine: Minimal improvement
- Visual Acuity: Normal or decreased
- Pupils: Normal
- Anterior Chamber: May have cell and flare
- Fluorescein: Fluorescein depicts branching pattern with terminal bulbs (HSV), branching with tapered ends (VSV), or diffuse fine keratitis (EKC).

Patients with HSV and VZV keratitis are treated with topical (trifluridine 1% q2h) and/or oral anti-virals (acyclovir, valacyclovir). Topical steroids may be added to reduce associated inflammation in treatment of VZV ophthalmicus, but this should only be done in consultation with ophthalmology and not without anti-virals. If treatment is initiated by the EP, a patient should follow up with ophthalmology within 24 hours. Otherwise, the patient should follow up with ophthalmology emergently (within 12 hours). The ophthalmologist must assess the depth of corneal involvement and associated sequelae of HSV (such as elevated IOP) to determine course of treatment.

## SUMMARY

It is important for the EP to perform a detailed history and physical examination in the patient who presents with an acute red eye. An assessment of the patient’s subjective symptoms along with a slit lamp examination focusing on a pattern of corneal fluorescein uptake or anterior chamber inflammation will significantly help narrow the diagnosis. While we did not specifically address patients with an abnormal lid examination, an EP should consider periorbital or orbital cellulitis, dacrocystitis, or blepharitis with associated eyelid erythema. Pain is an important distinguishing feature for the acute red eye; bacterial or viral keratitis, uveitis, AACG, corneal abrasion, or scleritis should be considered in patients with more than minimal pain or irritation. A patient’s pain will generally improve after instillation of topical anesthetics in processes isolated to the cornea, such as corneal abrasion and early viral or bacterial keratitis. Instillation of phenylephrine may help to distinguish between episcleritis and scleritis, since the redness of episcleritis typically improves after phenylephrine. The presence of cells and flare in the anterior chamber should raise suspicion for anterior uveitis or bacterial keratitis. Bacterial and viral keratitis and corneal abrasion/foreign body will have uptake on fluorescein examination. To summarize, patients with moderate or severe pain, photophobia, elevated intraocular pressure, anterior chamber inflammation, corneal epithelial defects with associated infiltrate, or decreased visual acuity should be referred urgently or emergently to ophthalmology.

## Supplementary Information



## Figures and Tables

**Figure 1:**
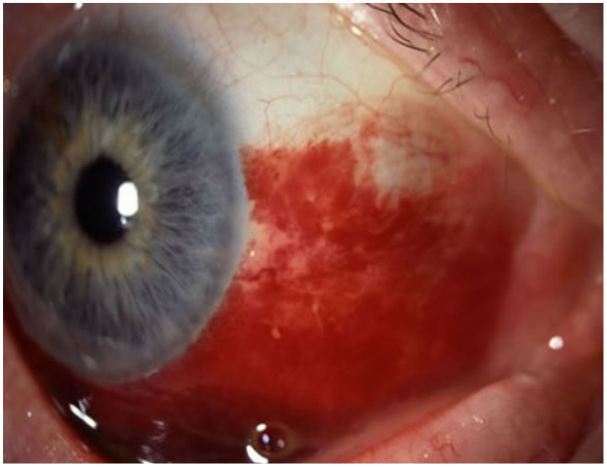
Subconjunctival hemorrhage. Image courtesy of Andrew Pearson, MA, MRCP.

**Figure 2:**
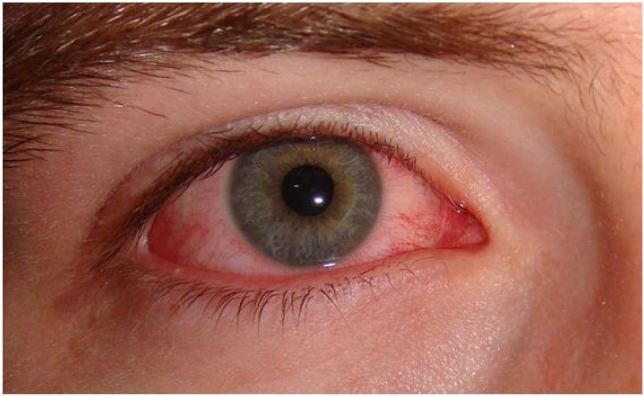
Acute viral conjunctivitis. Image courtesy of Wikimedia Creative Commons.

**Figure 3:**
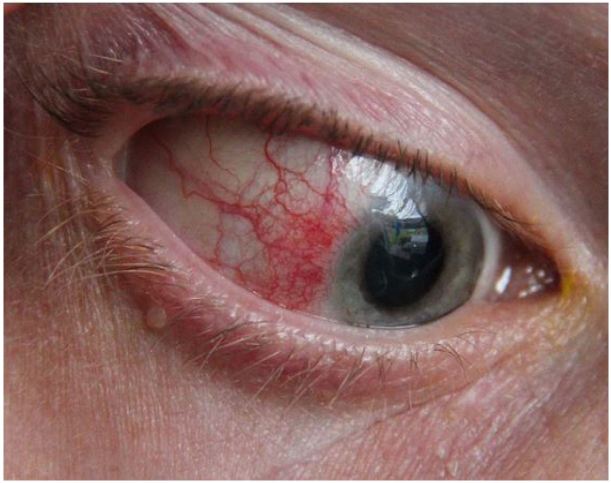
Episcleritis. Image courtesy of Asagan, Wikimedia Creative Commons.

**Figure 4:**
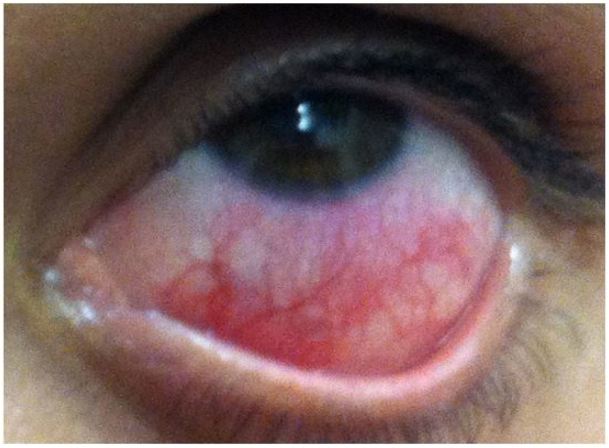
Anterior scleritis. Image courtesy of Marc Yonkers, MD, PhD.

**Figure 5:**
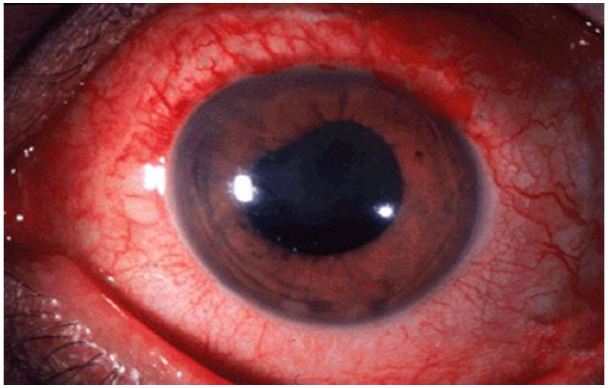
Anterior uveitis. Image courtesy of Jonathan Trove, MD, Wikimedia Creative Commons.

**Figure 6:**
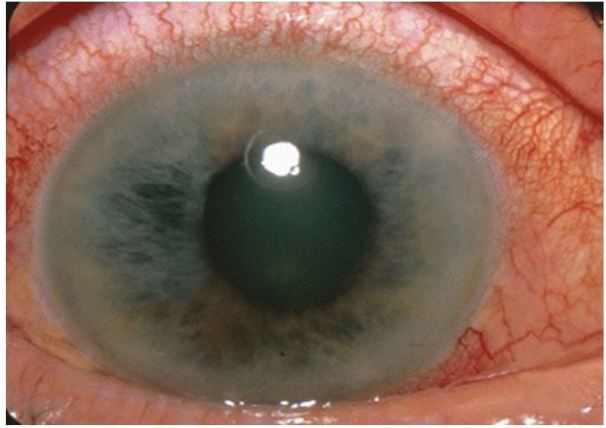
Acute angle closure glaucoma: note cloudy/“steamy” cornea and mid-position, fixed pupil. Image courtesy of Jonathan Trove, MD, Wikimedia Creative Common.

**Figure 7:**
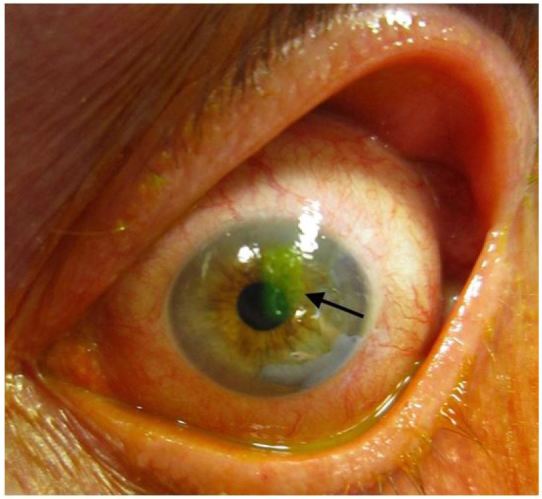
Corneal abrasion on fluorescein staining. Image courtesy of James Heilman, MD, Wikimedia Creative Commons.

**Figure 9:**
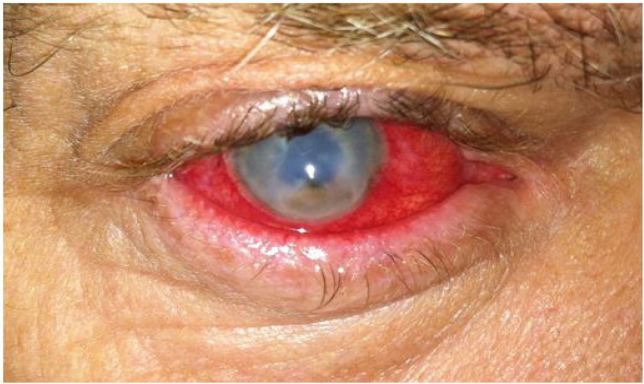
Endophthalmitis. Image courtesy of Marc Yonkers, MD, PhD.

**Figure 10:**
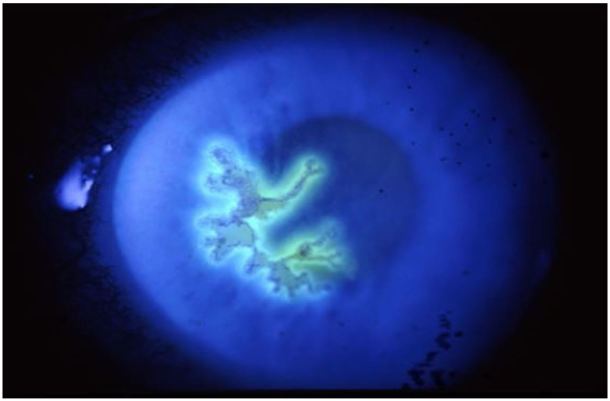
Viral keratitis (HSV keratitis). Image courtesy of Andrew Pearson, MA, MRCP.
