# Supplementary material for: Differentiating Urgent and Emergent Causes of Acute Red Eye for the Emergency Physician
Source: West J Emerg Med. 2017 Mar 3;18(3):509–17. doi: 10.5811/westjem.2016.12.31798 (PMC5391903; doi:10.5811/westjem.2016.12.31798)
Supplement: Supplementary file 1 [file wjem-18-509-s001.pdf]

Table 1: The differential diagnosis for the acute red eye in the emergency department

Condition	Definition	Risk factors and demographics	Associated symptoms	Pain? Photophobia? Proparacaine use? Phenylephrine use?	Redness is focal or diffuse?	Treatment? Ophthalmology referral (urgent vs. emergent)?	Anterior chamber (AC)? Pupils? Visual acuity (VA)? Fluorescein?	Additional Notes
**Subconjunctival Hemorrhage**<br>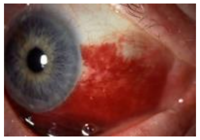<br>Image courtesy of Andrew Pearson, MA, MRCP	Heme under the conjunctiva, secondary to ruptured conjunctival blood vessel, which may be caused by coughing or straining[7]	Trauma, straining (coughing, sneezing, vomiting, Valsalva), conjunctivitis, chronic health conditions (diabetes, hypertension, coagulopathy)[8]	None	Pain: No<br><br>Photophobia: No<br><br>Proparacaine: N/A<br><br>Phenylephrine: No improvement of redness	Usually focal (in one sector) but may spread before it resolves	Treatment:<br>-Supportive care / topical lubrication<br><br>Referral: Primary care follow up as needed	AC: Clear<br><br>Pupils: Normal<br><br>VA: Normal<br><br>Fluorescein: No uptake	One should have high suspicion for globe rupture if there is trauma and/or diffuse or 360-degree bullous hemorrhage
**Conjunctivitis**<br>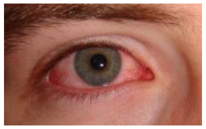<br>Image courtesy of Wikimedia Creative Commons	Infectious or non-infectious inflammation of the bulbar and palpebral conjunctiva[1]	Exposure to adenovirus or enterovirus, Staphylococcus aureus, Streptococcus pneumoniae, Haemophilus influenzae, Neisseria gonorrhea, Chlamydia trachomatis, Diphtheria, or history of allergy	Tearing, discharge, or associate viral symptoms	Pain: May have a mild burning sensation<br><br>Photophobia: No<br><br>Proparacaine: Improvement in irritation<br><br>Phenylephrine: Can have mild improvement of redness	Usually diffuse<br><br>Tarsal/palpebral conjunctiva SHOULD be involved in the redness	Treatment: Artificial tears 4-8x per day, consider topical antibiotics<br><br>Referral: Primary care follow up as needed	AC: Clear<br><br>Pupils: Normal<br><br>VA: Normal<br><br>Fluorescein: No uptake	Patients should not have photophobia (this should raise suspicion for a more serious condition)<br>Patients will commonly have discharge.<br><br>Viral conjunctivitis is highly contagious 1-2 weeks from onset. Recommend contact precautions and sterilization of patient encounter room after visit.
**Episcleritis**<br>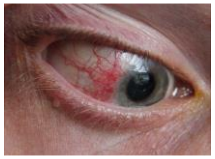<br>Image courtesy of: Asagan, Wikimedia Creative Commons	Inflammation of the episclera, which is the vascularized tissue between conjunctiva and sclera)[2]	-70% female[2]<br><br>-Fifth decade of life[2]<br><br>-One-third related to systemic autoimmune conditions[2]	Ocular redness without irritation or slight tenderness	Pain: Usually mild irritation, but chronic or nodular can have pain[2]<br><br>Photophobia: No<br><br>Proparacaine: May have improvement of irritation<br><br>Phenylephrine: Resolution of episcleral redness after 10-15 minutes	Most commonly focal in interpalpebral zone, can be diffuse	Treatment:<br>-Topical lubricants[15]<br>-Oral non-steroidal anti-inflammatory drugs<br>-Return precautions for symptoms of scleritis<br><br>Referral: Primary care follow up	AC: Clear<br><br>Pupils: Normal<br><br>VA: Normal<br><br>Fluorescein: No uptake	Most are idiopathic<br><br>Inflamed vessels can be moved with cotton tipped applicator
**Anterior Scleritis**<br>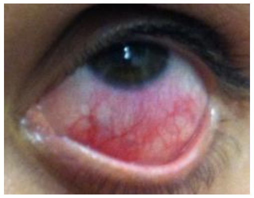<br>Image courtesy of Marc Yonkers, MD, PhD	Scleral inflammation, frequently associated with autoimmune systemic diseases[2]	50% autoimmune, systemic disease (rheumatoid arthritis, Wegener's granulomatosis), 4-10% infectious (Borrelia burgdorferi, tuberculosis, Nocardia asteroides, pseudomonas aeruginosa, Serratia marcescens, staphylococcus, streptoccus, syphilis, varicella zoster virus, CMV, aspergillus flavus, pseudallescheria boydii, protozoa acanthamoeba, toxoplasma gondii)[2]	Gradual onset, severe boring pain	Pain: Severe boring / piercing pain is typically worse at night and with extraocular movements, and may radiate to the face.[2,16]<br><br>Photophobia: Possible<br><br>Proparacaine: No improvement<br><br>Phenylephrine: No improvement in scleral redness (episclera may still improve)	May be diffuse or localized, depending on the type, sclera may have a typical bluish hue (visualizing uvea under thinned sclera)[17]	Treatment: Systemic treatment with NSAIDs or immunosuppressive therapy.[17] Eye shield if there is a perforation risk.<br><br>Referral: Emergent[8]	AC: Many will have associated anterior uveitis[2]<br><br>Pupils: Normal<br><br>VA: Normal or decreased, depending on the extent of disease<br><br>Fluorescein: Occasionally will have peripheral keratitis, more common in necrotizing form[2]	There are three forms of anterior scleritis: diffuse, nodular, and necrotizing.[2] Necrotizing scleritis usually causes the most severe pain and the worst outcome.<br><br>Inflamed vessels cannot be moved with cotton tipped applicator
**Anterior Uveitis/Iritis**	Inflammation of the uvea (iris, choroid, and ciliary body), causing redness and pain.	Idiopathic, systemic diseases (spondyloarthropathies), infectious (syphilis, tuberculosis, Lyme disease, toxoplasmosis, herpesviruses,	Pain, redness, photophobia, consensual photophobia, tearing, decreased vision	Pain: Yes<br><br>Photophobia: Yes<br><br>Proparacaine: No	Diffuse, pronounced at the limbus (ciliary flush)	Treatment:<br>-Topical steroids, dilating drops (to prevent scarring of iris to lens); do not start steroids without ophthalmology	AC: Cells and flare<br><br>Pupil: Constricted or irregular pupil	-If a specific diagnosis is suspected can start workup and treat underlying condition

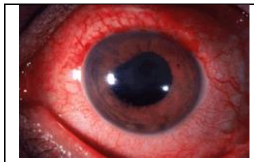<br><br>Image courtesy of Jonathan Trove, MD, Wikimedia Creative Commons		cytomegalovirus), drug induced (rifabutin, cidofovir, sulfas, moxifloxacin)[11]		improvement<br><br>Phenlyephrine: No improvement in redness		consultation.<br><br>Referral: Urgent (within 24 hours)	VA: Normal or decreased<br><br>Fluorescein: +/- (dendrites if HSV underlying cause)	
**Acute Angle Closure Glaucoma**<br><br>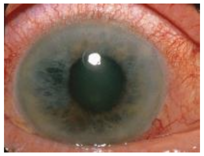<br><br>Image courtesy of Jonathan Trove, MD, Wikimedia Creative Commons	Closure (or narrowing) of the anterior chamber angle, causing elevated intraocular pressure and eventual optic nerve damage.[4,18]	-Increasing age<br>-Female gender (Three times more common)<br>-Asian ethnicity<br>-Shallow anterior chamber<br>-Hyperopia (farsightedness / "plus" glasses prescription), medication induced (topiramate or sulfa)[18]	Headache, nausea, vomiting, halos around lights, photophobia, blurred vision.	Pain: Yes, often with headache, nausea, and vomiting<br><br>Photophobia: Yes<br><br>Proparacaine: no improvement<br><br>Phenylephrine: should be avoided, may exacerbate attack[14]	Diffuse, with characteristic ciliary flush	Treatment:<br>Lower IOP in preparation for definitive treatment by ophthalmology (usually laser iridotomy)<br><br>IOP medications:<br>-Topical parasympathomimetics (pilocarpine 2%; avoid anything higher than 2%)<br><br>-Topical beta-blocker (0.5% timolol)<br>(caution in asthmatics, COPD patients, and patients with heart block)<br><br>-Carbonic anhydrase inhibitors (acetazolamide 500mg IV)<br>(Avoid in sickle cell patients and possibly in sulf- allergic patients)<br><br>-Alpha agonists (brimonidine 0.1%) (Avoid use in patients on monoamine oxidase inhibitors)<br>(Avoid apraclonidine as it can dilate and cause iris ischemia – has more alpha-1 adrenergic activity)<br><br>Referral: Emergent	AC: Shallow<br><br>Pupil: mid-sized or dilated, non-reactive pupil[14]<br><br>VA: Decreased<br><br>Fluorescein: No uptake	Elevated IOP, can be > 60 mm Hg<br><br>Can have permanent vision loss within hours
**Corneal Abrasions/**<br>**Corneal Foreign Bodies**<br><br>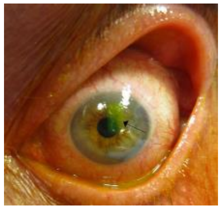<br><br>Image courtesy of James Heilman, MD Wikimedia Creative Commons	Defect (or foreign body) of the corneal epithelium, causing irritation, pain, tearing, and photophobia[3]	-History of trauma, contact lens use, male gender, young adults, construction or manufacturing job without eye protection (foreign body)	-Photophobia, watering, tearing, foreign body sensation	Pain: Yes, usually lasting less than 24- 48 hours[3]<br><br>Photophobia: Yes<br><br>Proparacaine: Improves symptoms<br><br>Phenylephrine: Redness improves	Diffuse	Treatment:<br>Corneal abrasion:<br>-Lid eversion to exclude foreign body<br>-Lubricating ointment or drops<br>-Topical antibiotics (polymixin B/trimethoprim or polysporin; quinolones for contact lens wearers)<br><br>Corneal foreign body:<br>-Removal by ophthalmology or ED physician, depending on location, size, and presence of rust ring<br>-Topical antibiotics<br><br>Referral: Contact lens wearers: urgent (however, emergent if concern for corneal ulcer– presence of ulcer or hypopyon, pain not improving within 24 hours)	AC: Clear<br><br>Pupil: Normal<br><br>VA: May be decreased if the abrasion/FB is in the visual axis, however, this should raise concern for alternative diagnosis<br><br>Fluorescein: Uptake at the corneal abrasion or foreign body	
**Bacterial or Fungal Keratitis/Corneal Ulcer**	Corneal epithelial defect with stromal haze due to microorganisms[5]	-Contact lens use[19]<br>-Agricultural work[10]<br>-Eye trauma<br>-Use of corticosteroids<br>-Systemic diseases (diabetes)<br>-Prior ocular surgery<br>-Chronic ocular surface disease[10]<br><br>-Microorganisms (Streptococcus	Significant pain, tearing, and discharge	Pain: Yes<br><br>Photophobia: Yes<br><br>Proparacaine: May improve pain<br><br>Phenylephrine: May improve redness	Diffuse	Treatment: Fortified topical antibiotics<br><br>Referral: Emergent	AC: Possible cells and flare<br><br>Pupil: Normal<br><br>VA: Decreased if involving visual axis<br><br>Fluorescein: Large uptake over area of ulceration	

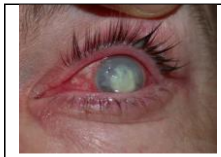<br>Image courtesy of Andrew Pearson, MA, MRCP		pneumoniae, staphylococcus aureus, mycobacterium fortuitum, M. chelonae, Nicardia spp., Pseudomonas aeruginosa, Enterobactriaceae, Moraxella, Haemophilus, Neisseria gonohoeae; fungi: Fusarium, Aspergillus, Curvularia, Candida, Cryptococcus; protozoa: Acanthamoeba)						
**Endophthalmitis**<br>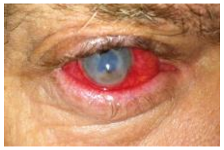<br>Image courtesy of Marc Yonkers, MD, PhD	Bacterial or fungal) infection involving the vitreous and/or aqueous humor[9]	-Eye surgery (cataract surgery has 0.1% risk)<br>-Penetrating ocular trauma<br>-Corneal infection<br>-Intravitreal injections<br>-(Fungal) hospitalization with central venous access, total parenteral nutrition, or broad spectrum antibiotics[9]<br><br>Microorganisms: Coagulase-negative staphylococcus, viridans strep (post-procedure), Bacillus cereus (post-traumatic), staphylococcus, streptococcus, candida (endogenous), klebsiella pneumoniiae (East Asia, associated with liver abscess)	Pain and decreased vision	Pain: Yes<br><br>Photophobia: Yes<br><br>Proparacaine: No improvement<br><br>Phenylephrine: May have some improvement	Diffuse	Treatment:<br>-If vision "hand motion" or better, then intravitreal injection of antibiotics<br>-If vision "light perception" or worse, then surgery<br><br>Referral: Emergent	AC: Commonly associated with hypopyon[9]<br><br>Visual acuity: Decreased<br><br>Pupils: Severe cases may have afferent pupillary defect[20]<br><br>Fluorescein: May diagnose the inciting penetrating injury or corneal abrasion	Usually occurs 2-7 days post-operatively or 12-24 hours after trauma
**Viral Keratitis**<br>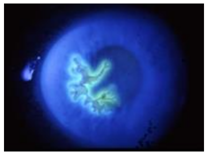<br>Image courtesy of Andrew Pearson, MA, MRCP	Corneal inflammation caused by herpes simplex, varicella zoster, or adenovirus (epidemic keratoconjunctivitis, EKC) characterized by pain, tearing, photophobia, and corneal epithelial defects.	-HSV-1 (most commonly), HSV-2, varicella zoster virus,, adenovirus 8, 19, 37[21]	VZV: Hutchinson's sign (vesicular lesion on the nose, VZV), although this is not sensitive or specific[22]<br><br>HSV: vesicular rash. Can have high IOP in HSV induced uveitis	Pain: Yes<br><br>Photophobia: Yes<br><br>Proparacaine: Improvement in pain<br><br>Phenylephrine: May improve redness	Diffuse, with ciliary flush	Treatment:<br>-Topical (trifluridine 1% q2h) and/or oral anti-virals (acyclovir, valacyclovir)<br>-Topical steroids may be added in treatment of VZV ophthalmicus, but this should ONLY be done in consultation with ophthalmology<br><br>Referral: Emergent	AC: Possible cells and flare<br><br>VA: Normal or decreased<br><br>Pupils: Normal<br><br>Fluroescein: HSV: branching pattern with terminal bulbs; VZV: branching with tapered ends; EKC: diffuse fine keratitis	
